# Knowledge on exotic mosquitoes in Germany, and public acceptance and effectiveness of Bti and two self-prepared insecticides against *Aedes japonicus japonicus*

**DOI:** 10.1038/s41598-020-75780-5

**Published:** 2020-11-03

**Authors:** Friederike Reuss, Aljoscha Kreß, Markus Braun, Axel Magdeburg, Markus Pfenninger, Ruth Müller, Marion Mehring

**Affiliations:** 1 Senckenberg Biodiversity and Climate Research Centre (SBiK-F), Georg-Voigt-Straße 14-16, 60325 Frankfurt am Main, Germany; 2 Institute of Occupational Medicine, Social Medicine and Environmental Medicine, Goethe University Frankfurt, Theodor-Stern-Kai 7, 60590 Frankfurt am Main, Germany; 3 LOEWE TBG (Translational Biodiversity Genomics), Senckenberganlage 25, 60325 Frankfurt am Main, Germany; 4 Institute of Organismic and Molecular Evolution (iOME), Johannes Gutenberg University, Gresemundweg 2, 55128 Mainz, Germany; 5 Institute of Tropical Medicine, Nationalestraat 155, 2000 Antwerp, Belgium; 6 ISOE - Institute for Social-Ecological Research, Biodiversity and People, Hamburger Allee 45, 60486 Frankfurt am Main, Germany

**Keywords:** Infectious diseases, Ecological epidemiology, Freshwater ecology, Public health, Natural products, Climate-change ecology

## Abstract

Mosquito-borne diseases are a continuous challenge to public health. To prevent transmission, Integrated Vector Management (IVM) applies preventive, control, and communicational strategies that should be feasible, environmentally benign, and sustainable. IVM shows higher efficiency when being supported by local communities. Accordingly, we applied a social-ecological approach to identify the public acceptance of control measures and effectiveness of Eurocent coins containing copper, clove essential oil (EO) and *Bacillus thuringiensis israelensis* (Bti). We performed field and laboratory experiments to demonstrate the toxicity of alternative substances against *Aedes japonicus japonicus.* In expert interviews, we asked for (1) knowledge on exotic mosquitoes in Germany, (2) potential chances of alternative substances in future mosquito control, and (3) their needs for further clarification before application. We assessed potential users’ (4) awareness of exotic mosquitoes and (5) willingness to apply the substances. Self-prepared copper coins and EO were clearly preferred by potential users over Bti. However, 100% mortality of the sensitive first stage could not be reached with the number of ten 5-Eurocent coins showing limited toxicity. Clove EO was shown to work as oviposition deterrent and larvicide with a LC_50_ of 17 mg l^−1^ (95% CI: 15–19 mg l^−1^). This study shows the importance of potential users’ perspectives in IVM and the need for authorised insecticides.

## Introduction

In the presence of increasing global connectivity and climate change, mosquito (Diptera: Culicidae) species, which are of public health importance due to their bloodsucking behaviour, shift or expand their distribution ranges and establish in yet unaffected areas^1,2^. Since many exotic mosquitoes are also competent vectors of pathogens that can cause severe diseases in humans or their livestock^3^, these introductions are often closely monitored^4^. However, in order to actually prevent disease transmission, an effective control of the vector (mosquito) population is necessary^5,6^.

Vector population suppression can be achieved by insecticide application of chemical or biological agents^7^, genetic measures^8^, host manipulations^9^ or continuous source reduction of breeding habitats^10^, although each of those approaches have their specific drawbacks: Insecticides can affect non-target organisms^11–13^ and insecticide resistance build-up^14^ complicates vector control through chemical agents^15,16^. Genetic measures involve the release of genetically modified organisms which is controversially debated^17,18^. Host manipulations, especially through *Wolbachia pipientis*-induced sterility, have to be monitored post release^19^. Source reduction is time- and resource-intense^20^.

One integral part of Integrated Vector Management (IVM) is the synergistic use of a combination of these different control tools. Also, only evidence-based methods for surveillance, monitoring and evaluation should be applied, interdisciplinary collaboration between professional disciplines and within the health sector should be involved, and local stakeholders should be mobilised and engaged in IVM^21,22^.

Therefore, the public perception of mosquito population control measurements is of high importance within the IVM framework. A further argument to engage local stakeholders is that mosquitoes such as *Aedes japonicus japonicus* Theobald, 1901 (the Asian bush mosquito) and *Ae. albopictus* Skuse, 1894 (the Asian tiger mosquito) often breed in urban and suburban areas and mainly in man-made containers, e.g., rain barrels, vases, water collection in plastic covers, plastic toys, car tires, flower pots^23^. Such small breeding sites are found in both public and private space^24^, but the extension of control campaigns to private grounds is challenging because access permission needs to be given^25^.

In previous studies, communities were encouraged to take part in IVM by applying personal protection through repellent use or larval source reduction with varying success^26,27^. Also, the application of larvicides on private property was shown to be essential to reduce *Ae. albopictus* egg densities in Italy^28^, the USA^24^, and Spain^25^. Consequently, the possibility of an insecticide application directly implemented by private persons on their properties should be assessed. A prerequisite for this form of community engagement is the acceptance of the insecticide by the users.

To develop effective insecticide tools with high societal acceptance and support to implement them, we chose *Ae. j. japonicus* as study organism, since it is one of the *Aedes*-mosquitoes invasively spreading across Europe^29^ and North America^30^ and its extensive distribution area in Germany^31^. The distribution is likely to expand through climate change and this species will potentially form more generations per year in the future^32^. The species’ potential to transmit pathogens like Japanese encephalitis^33^, dengue, chikungunya^34^ or West Nile viruses^35^ may lead in the future to the necessity to control or eradicate vector populations thus minimising pathogen transmission to the human population. In 2019, there was the first reported mosquito-to-human West Nile virus transmission in Germany showing that the virus is already present in the country^36^. Until now, *Ae. j. japonicus* has been operationally controlled by means of *Bacillus thuringiensis israelensis* (Bti) in Belgium^37^ and modelling based on lifetable data showed that containment of the species can be achieved by control measures targeting different stages^38^.

In the present study, we assessed the effectiveness of different substances as control agents against this exotic mosquito species and asked for the willingness of local people to apply these agents on their private properties. Initially, (1) we evaluated the efficiency of substances, known from the literature for their mosquito-repellent or toxic activities, against eggs and larvae of *Ae. j. japonicus*. Essential oils (EOs) are among the potential, plant-based control agents with known mosquito-repellent or -toxic activities^39,40^. Besides EOs, we also screened soap^41^, two leaf extracts^42,43^, copper^44–46^, and the insecticides Bti^37,47^ and pyrethrum^48^.

(2) We evaluated the acceptance of the highly efficient clove EO, and Eurocent coins by local experts and identified needs for clarification. Based thereupon, (3) we ruled out potential application limitations for the use of Eurocent coins as copper sources, e.g., copper solubility in water and toxic effect on cut flowers. In addition, (4) we assessed the willingness of potential users to implement the respective control agents, Eurocent coins, EOs, and Bti tablets.

## Methods

### Field experiment on the oviposition deterrent effect of eleven substances

The potential oviposition deterrent effect of eleven substances (Table 1) was determined in a field experiment clustered in early season (from 17 June to 2 July 2014) and peak season (from 27 July to 2 September 2014). The monthly mean temperature measured at the nearby weather station in Lahr (Deutscher Wetterdienst station no. 10805) increased from June 2014 (19.3 °C) to July (19.8 °C) and decreased in August (17.8 °C) and September (16.1 °C). The study site was a garden in a rural area of Biberach (Baden), Germany. There, *Ae. j. japonicus* breeding has been documented every year since 2012. The surrounding vegetation consisted of either lawn or unplanted mulched soil, or ground covered with ivy or berry bushes without understory vegetation. At three sites in the garden, 16 black 200 ml plastic cups filled with 150 ml of eleven substances including solvents (Table 1) and respective solvent controls (one ethanol, four water) were set up in a block design. The cups in each block were placed in close proximity (about 10 cm apart) to allow approaching female mosquitoes to a better overview and distinguish the containers offered for oviposition. For this purpose, cups were carved to prevent them from tilting over. A wooden tongue depressor was added to each cup to serve as a stick and additional surface assisting oviposition. Ten % ethanol was solved in deionised water and used as solvent for clove EO, mint EO, eucalyptus EO, lavender EO, pyrethrum, and tea tree EO. Deionised water was used as solvent for Bti, copper band, Japanese beautyberry, soap, and walnut.

**Table 1:** Substances added to potential breeding habitats in the field experiment to test for oviposition deterrent effects against *Ae. j. japonicus*.

Substance	Product (manufacturer)	Concentration
Bti (*Bacillus thuringiensis israelensis*)	StechmückenFrei (Neudorff, Emmerthal, Germany)	1.5 µl l^−1^
Clove (*Eugenia caryophyllata*)	Clove stem oil (Dagmar Köhler, Alpen, Germany)	1 g in 150 ml deionised water
Copper band	FloraSelf Kupfer Schneckenband (Hornbach, Bornheim, Germany)	Piece of 3 cm by 3 cm
Deionised water	–	Pure
Ethanol	Ethanol absolute (Merck, Darmstadt, Germany)	10%
Eucalyptus (*Eucalyptus radiat*a)	Eukalyptusöl radiata (Dagmar Köhler, Alpen, Germany)	1 g in 150 ml deionised water
Japanese beautyberry leaves (*Callicarpa japonica*)	Dried, grinded leaves collected in Frankfurt am Main, Germany	1 g l^−1^
Lavender (*Lavandula angustifolia*)	Lavendelöl Mont Blanc (Dagmar Köhler)	1 g in 150 ml deionised water
Mint (*Mentha arvensis* var. *javanesa*)	Japanisches Heilpflanzenöl (Das gesunde Plus, dm, Karlsruhe, Germany)	1 g in 150 ml deionised water
Pyrethrum (*Chrysanthemum* sp.)	Pyrethrum extract Pestanal (Sigma-Aldrich, Steinheim, Germany)	0.1 mg l^−1^
Soap	Frosch Spülmittel (Werner and Mertz, Mainz, Germany)	1 g l^−1^
Tea tree (*Melaleuca alternifolia*)	Teebaumöl EuAB 5.00 (Dagmar Köhler)	1 g in 150 ml deionised water
Walnut leaves (*Juglans regia*)	Dried, grinded leaves collected in Biberach (Baden), Germany	1 g l^−1^

Product usage specifications were followed for Bti. All other products were not intended to be used as mosquitocides in water. Therefore, no usage specifications were followed.

Oviposition was measured by counting the eggs on the wooden sticks under a stereo microscope or with the help of a magnifying glass (3.5-fold magnification) on the last day of the respective sampling period. To ensure that only eggs of *Ae. j. japonicus* were counted, egg rafts of *Culex* or *Culiseta* species were not taken into account and removed at the field site, larval hatching was stimulated by flooding the eggs with deionised water. Then, larvae were reared until adult emergence and hatched imagines were morphologically identified.

Eggs were counted on the oviposition stick and inner wall of cups on the last day of the respective time period and eggs, water as well as cups were taken to the laboratory to check for the presence of other mosquito species. For that, aquatic stages and eggs were reared to adults and morphologically checked. Substances and cups were refreshed on 17 June and 27 July.

The oviposition activity index (OAI) was calculated according to^49^ as

1 $$\mathrm{OAI}=\frac{(\mathrm{NT}-\mathrm{NS})}{(\mathrm{NT}+\mathrm{NS})}$$

with NT being the number of eggs in the treatment and NS being the number of eggs in the respective solvent control (deionised water or ethanol). The percentage of effective repellency (ER%) is the percentage amount of laid eggs in the treatment (NT) related to the number of eggs in the control (NS):

2 $$ER\%=\frac{NS-NT}{NS} \times 100$$

### Laboratory experiments with copper Eurocent coins

Laboratory experiments on the toxic effects of copper-containing Eurocent coins on larvae of *Ae. j. japonicus* and cut flowers were conducted. For toxicity testing, pre-tests were necessary. In copper experiment 1, the solubility of copper(I)-ions (Cu^+^) and copper(II)-ions (Cu^2+^) from Eurocent coins was tested in three different types of water. In copper experiment 2, Cu^2+^-concentrations resulting from 1-, 2- and 5-Eurocent coins were recorded. In copper experiment 3, the acute toxicity of Eurocent coins on *Ae. j. japonicus* larvae was assessed. In copper experiment 4, specifically addressing concerns from expert interviews, the acute toxicity of Eurocent coins on cut flowers was assessed.

For copper experiment 1, we compared the copper solubility in tap water, deionised water, and rainwater. The latter had been collected from 20 to 23 March 2018 in Frankfurt am Main, Germany, and filtered through a coffee filter. All three types of water were brought to 16 °C before starting the experiment on 26 March 2018. For that, transparent 1 l plastic cups were filled with 800 ml of each type of water (n = 3) and one 2-Eurocent coin was added. At the first day (experimental onset) and days 2, 3, 4, 8, 15 and 30, the following water parameters were recorded: Cu^+^-, Cu^2+^-concentration (semi-quantitative colorimetric copper test, Merck Millipore, Darmstadt, Germany), pH-value, and conductivity (sensors; WTW, Weinheim, Germany). During the experiment, the ambient temperature was set to 16 °C ± 1 °C. Cu^+^- and Cu^2+^-concentrations on day 30 between water types were statistically compared by non-parametric Mann–Whitney U tests in GraphPad Prism version 5.00 for Windows (GraphPad, San Diego, California, USA). Two-tailed *p* values are given.

For copper experiment 2, ten experimental treatments were set up: one, two or three coins of 1-, 2- or 5-Eurocent coins plus a control without copper treatment. The coins were added to 800 ml deionised water in transparent 1-l plastic cups and incubated at 17 °C ± 1 °C. The concentration of Cu^2+^ and conductivity were recorded one day before the addition of coins, and at days 4, 8, 16, and 32 after experimental onset.

For copper experiment 3, *Ae. j. japonicus* eggs were collected in Lorch am Rhein, Germany, from 3 to 10 July 2018 using 2-l black plastic cups equipped with pressboard sticks as oviposition substrate. The following Cu^2+^-concentrations were set: 0.00 (no coin), 0.2 (1 × 1-cent), 0.4 (2 × 1-cent), 0.6 (2 × 5-cent), 0.9 (3 × 5-cent), 1.2 (4 × 5-cent), and 2.5 (10 × 5-cent) mg l^−1^. Coins were incubated for 8 days before the onset of the experiment to reach desired concentrations (estimated due to results from copper experiment 2). Coins remained in the cups during the experiment. Larval hatch was triggered by placing the oviposition sticks in deionised water on 17 July 2018. The next day, an acute toxicity test was conducted with larvae younger than 24 h. One hundred larvae per copper concentration (20 larvae per cup, five cups) were tested at 25 °C. After 24 h, living larvae were counted and mortality was calculated considering moribund larvae as dead. Cu^2+^-concentrations were log-transformed and lethal concentration (LC) values LC_10_, LC_50_ and LC_90_ were calculated using non-linear regression models with the least squares method in GraphPad Prism. LC values represent the lethal concentrations at which 10%, 50% and 90% of the larvae die, respectively.

In copper experiment 4, the effect of copper coins on cut flowers was measured. Therefore, transparent 1-l plastic cups were filled with a 1:1 mixture of tap water and rainwater, and three cut roses were inserted. No coin or a 2-Eurocent coin were inserted and the status of cut roses were photographically documented on days 0, 2, 4, 8, 11 and 16 after experimental onset. The experiment was conducted at 17 °C ± 1 °C and about 800 lx artificial light. Additionally, cups were placed on a window ledge so that plants experienced a circadian rhythm with natural sunlight.

### Laboratory experiment on the toxic effect of clove EO

To test the acute larval toxicity of clove EO (oil of cloves ≥ 80%, natural, rect.; Carl Roth, Karlsruhe, Germany), eggs were collected in Hadamar and Dornburg, Germany, from 13 to 27 June 2018. Larval hatch was stimulated by addition of deionised water on 1 July 2018 and one day later, the experiment was started using larvae younger than 24 h. Larvae were exposed to 0, 10, 20, 40, 80, 160, 320 mg l^−1^ clove EO at 25 °C. Five cups per concentration per site were used containing 20 larvae each. This corresponds to 100 larvae per concentration per site and 1,400 larvae in total. After 24 h, moving larvae were counted and the mortality calculated and analysed as described for copper experiment 3. Nonlinear regressions were made as described for copper and data of both egg sampling locations were compared by unpaired, two-tailed t-tests in GraphPad Prism. We did not do a chemical analysis of active ingredients of the used clove EO but the certificate of analysis provided by the supplier names a concentration of 82.1% eugenol in the batch log.

### Expert interviews

Twenty qualitative-explorative expert interviews were conducted via telephone in October and November 2017 with heads of green area offices or building yards, local authorities responsible for cemetery maintenance, cemetery administrators, municipal/communal gardeners, and gardeners from private cemetery nurseries commissioned to maintain graves in the study area. Interview partners are named “experts” from here on. The interviews lasted between 10 and 20 min. They were recorded as audio files, transcribed and analysed using the qualitative data analysis software MaxQDA. The selection of the interview partners followed a purposive sampling design representing municipalities of different sizes: < 8000 inhabitants, 20,000–40,000 inhabitants, and 150,000–400,000 inhabitants. The municipalities are located in the federal states of Hesse, Rhineland-Palatinate, Baden-Württemberg and North Rhine-Westphalia. In most of these municipalities *Ae. j. japonicus* has already been found in one of the cemeteries. The interview contents can be found in Supplement S1. For the analysis of this article, only the questions on the evaluation of the respective measures as well as needs for clarification were considered. This study was approved by the Ethics Committee of the ISOE—Institute for Social-Ecological Research.

### Telephone survey

In September and October 2018 a standardized Computer Aided Telephone Interviews (CATI) survey was conducted. The selection of the interview partners followed a random sampling design representing the groups of gardeners and grave attendants in the study region. The sampling approach was a representative sampling approach. In total 257 interviews with users of an allotment garden (gardeners) and 150 interviews with cemetery visitors who maintain a grave (grave attendants) were conducted. The CATI partners were chosen according to the following criteria: Gardeners: manage a garden either directly by their house or in an allotment garden; use a rain barrel and/or flower tubs with coasters in which water accumulates and/or have a pond in the garden. Grave attendants: take care of the grave themselves on a regular basis and have flower vases, flower bowls with saucers or other containers in which water can be collected on the grave, at least temporarily. CATIs were carried out in the federal states of Hesse, Baden-Württemberg and Rhineland-Palatinate of Germany, which are currently most severely infested by *Ae. j. japonicus*. Each CATI lasted about 10 to 15 min. The CATI questionnaire can be found in Supplement S2. For the analysis of this article, only the questions on the awareness of the new mosquitoes, from where they have heard about it, knowledge about *Ae. j. japonicus* and *Ae. albopictus*, as well as their willingness to implement the respective measures were considered.

This study was approved by the Ethics Committee of the ISOE—Institute for Social-Ecological Research.

### Human participants

The expert interviews and telephone surveys were carried out in accordance with the ethical guidelines and regulations of the ISOE—Institute for Social-Ecological Research and approved by the Ethics Committee of the ISOE—Institute for Social-Ecological Research. We confirm that informed content was obtained from all interviewees or from a parent and/or legal guardian.

## Results

### Oviposition deterrence of EOs and other substances

In the field experiment, no other mosquito taxa than *Ae. j. japonicus* were found. In total, 2221 eggs were found in the experimental setup. During June/July, 442 eggs per week were found which corresponds to 883 eggs during the whole period of time (Supplement S3). From July to September, 1338 eggs were found which equals 149 eggs per week. For the first period of time, most eggs were found in a water control (sum of total eggs in three blocks: 163) followed by the walnut treatment (123 eggs). No eggs were laid in the clove EO treated cups. From July to September, 159 eggs (total sum of three blocks) were oviposited in the teatree EO cups, followed by walnut (148 eggs) and water (147 eggs). The best oviposition deterrent effect was reached with clove EO, followed by lavender EO (Table 2). Both showed a reduction in laid eggs during both experimental periods. Clove EO showed a reduction of more than 80% compared to the respective control. Copper applied as copper band showed no oviposition deterrent effect.

**Table 2:** Oviposition activity index (OAI) and percentage of effective repellency (ER%) of substances tested in the field survey.

	June/July		July–September		Mean of seasonal replicates	
	OAI	ER%	OAI	ER%	OAI	ER%
Walnut	+ 0.12	− 26.48	0.33	− 100.00	+ 0.16	− 37.11
Copper band	+ 0.01	− 1.80	0.03	− 6.76	+ 0.01	− 2.52
Soap	+ 0.00	− 0.77	− 0.33	50.00	− 0.03	+ 6.57
Bti	− 0.30	+ 46.53	0.27	− 75.68	− 0.17	+ 28.85
Japanese beautyberry	− 0.53	+ 69.15	0.11	− 25.68	− 0.38	+ 55.44
Mint	+ 0.06	− 13.79	0.19	− 46.88	+ 0.11	− 24.68
Teatree	− 0.71	+ 82.76	0.43	− 148.44	− 0.03	+ 6.68
Eucalyptus	− 0.18	+ 31.03	0.10	− 21.88	− 0.07	+ 13.62
Pyrethrum	− 0.81	+ 89.66	0.30	− 87.50	− 0.19	+ 31.36
Lavender	− 0.87	+ 93.10	− 0.29	45.31	− 0.63	+ 77.38
Clove	− 1.00	+ 100.00	− 0.86	92.19	− 0.95	+ 97.43

Negative OAIs and positive ER% values indicate an oviposition deterrent activity compared to the respective controls. The raw data as number of eggs and number of eggs per week can be found in the Supplement S3.

### Solubility and toxicity of Eurocent coins (copper experiments 1–4)

**Figure 1:**
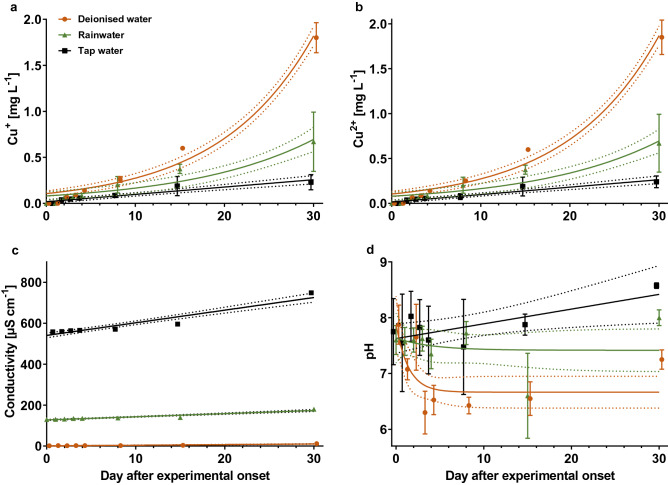
Solubility of copper ions originating from 2-Eurocent coins in three different water types and measurements of conductivity and pH (copper experiment 1): (**a**) Measured Cu^+^-concentration during the course of the experiment; (**b**) Cu^2+^-concentration; (**c**) conductivity; (**d**) pH-value. Symbols represent mean values and standard deviations are shown as error bars. For nonlinear regressions (solid lines), the 95% confidence intervals are displayed as dotted lines.

No copper ions were detected in the three water types before adding the Eurocent coins. In copper experiment 1, the highest Cu^+^- and Cu^2+^-concentrations were found in deionised water followed by rainwater and tap water (deionised water vs. tap water and rain water *p* = 0.02). During the experiment, the concentrations raised exponentially (Supplement S4) with highest values at the end at day 30 (Fig. 1). Lowest conductivity was measured in deionised water and increased from rainwater to tap water (Fig. 1c). While pH is almost constant in rainwater during the experiment, it decreases in deionised water and increases in tap water (Fig. 1d). The equations for regressions are shown in Supplement S4.

Photographic documentation made on experimental day 30, shows that coins can be returned into the circulation of money after approximately one month of usage as they do not show signs of erosion albeit occasional formation of rust was observed from day 8 onwards (Supplement S5).

**Figure 2:**
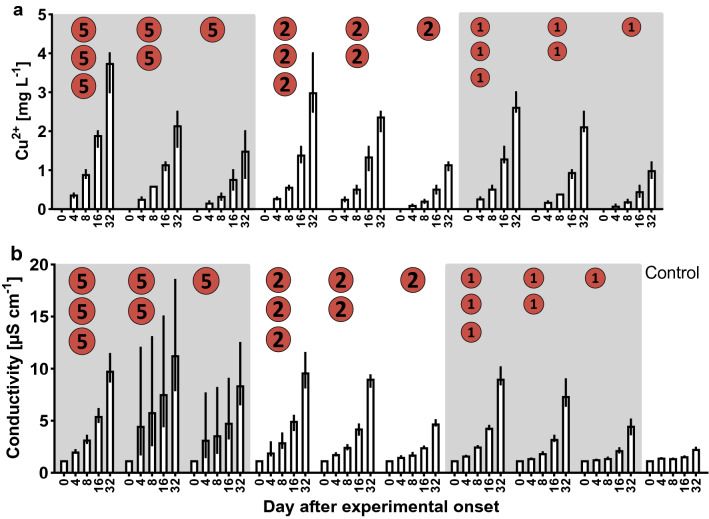
Solubility of Cu^2+^ from different Eurocent coins (copper experiment 2): (**a**) Measured Cu^2+^-concentration during the course of the experiment; (**b**) conductivity. Mean values with min to max range (error bars) are shown.

During copper experiment 2, the solubility of copper ions from different combinations of Eurocent coins were proven (Fig. 2a). Highest copper concentration and the highest conductivity were found after 32 days in all assays. Cu^2+^-concentrations and conductivity were higher with higher denomination of coins and incubation duration (Fig. 2). The highest copper concentration was 3.75 mg l^−1^ in the assay with three 5-Eurocent coins, the highest conductivity was 11.3 µS cm^−1^ in the assay with two 5-Eurocent coins.

Mean values of Cu^2+^ of Eurocent coin treatments in copper experiment 2 (Table 3) were used for calculations of LC values in copper experiment 3.

**Table 3:** Mean values of Cu^2+^-concentrations per treatment group and day after experimental start.

Treatment	Cu^2+^-concentration in mg l^−1^			
	Day 4	Day 8	Day 16	Day 32
3 × 5-Eurocent	0.38 ± 0.05	0.90 ± 0.12	1.90 ± 0.20	3.75 ± 0.50
2 × 5-Eurocent	0.26 ± 0.03	0.60 ± 0.00	1.15 ± 0.10	2.15 ± 0.44
1 × 5-Eurocent	0.17 ± 0.02	0.34 ± 0.08	0.78 ± 0.21	1.50 ± 0.50
3 × 2-Eurocent	0.29 ± 0.03	0.58 ± 0.05	1.40 ± 0.23	3.00 ± 0.71
2 × 2-Eurocent	0.26 ± 0.05	0.53 ± 0.10	1.35 ± 0.30	2.38 ± 0.25
1 × 2-Eurocent	0.11 ± 0.02	0.22 ± 0.04	0.53 ± 0.10	1.15 ± 0.10
3 × 1-Eurocent	0.28 ± 0.03	0.53 ± 0.05	1.30 ± 0.20	2.63 ± 0.25
2 × 1-Eurocent	0.19 ± 0.02	0.40 ± 0.00	0.95 ± 0.10	2.13 ± 0.25
1 × 1-Eurocent	0.09 ± 0.02	0.20 ± 0.05	0.46 ± 0.16	1.00 ± 0.23

Mean values ± standard deviations are given.

LC values for Cu^2+^-ions from Eurocent coins are LC_10_ = 0.09 mg l^−1^ (95% CI: 0.05–0.18 mg l^−1^), LC_50_ = 0.53 mg l^−1^ (95% CI: 0.42–0.66 mg l^−1^), LC_90_ = 3.04 mg l^−1^ (95% CI: 1.69–5.46 mg l^−1^). A 100% mortality of larvae has never been reached with the addition of up to ten 5-Eurocent coins to the test vessel.

Copper experiment 4 showed that the appearance of cut roses is not affected by copper coins. Flower heads have drooped at day 8, and no difference between the treated and untreated group could be observed (Supplement S6, S7).

### Larval toxicity of clove EO

LC values are LC_10_ = 7.93 mg l^−1^ clove EO with a 95% confidence interval (95% CI) ranging from 6.47 to 9.71 mg l^−1^, LC_50_ = 18.36 mg l^−1^ (95% CI: 16.84–20.02 mg l^−1^), and LC_90_ = 42.52 mg l^−1^ (95% CI: 34.98–51.67 mg l^−1^) for Hadamar and LC_10_ = 5.15 mg l^−1^ (95% CI: 2.81–9.44 mg l^−1^), LC_50_ = 16.58 mg l^−1^ (95% CI: 12.83–21.43 mg l^−1^), and LC_90_ = 53.34 mg l^−1^ (95% CI: 29.97–98.20 mg l^−1^) for Dorndorf. Global comparison of fits showed that no significant differences between datasets were found (F = 0.6992, *p* = 0.5010). Thus, combined LC values for both locations were LC_10_ = 6.15 mg l^−1^ (95% CI: 4.51–7.97 mg l^−1^), LC_50_ = 17.08 mg l^−1^ (95% CI: 15.10–19.20 mg l^−1^), and LC_90_ = 47.43 mg l^−1^ (95% CI: 38.53–60.49 mg l^−1^). Larval mortality of 100% was observed in cups with 80 mg l^−1^ and higher concentrations. Regression equations are shown in Supplement S8.

### Expert interviews

The Eurocent coin method was perceived to be the most promising and convincing control measure (Table 4). According to the experts, a potential measure needs to be cheap and easy to apply. Thus, they favored the copper coin over the use of EO. The application of Bti was largely rejected from the experts. The main reason mentioned by the experts was the perception that Bti is a chemical /not natural way of combating mosquitoes. In particular, they expressed the fear that other organisms might be negatively affected. This aspect of potential side effects also hold true for the other proposed control measures. In the expert interviews, it became clear that environmental aspects (impact on environment and organisms) play a serious role for the experts. Consequently, experts indicated potential side effects to be considered when applying the respective control measure. In addition, further information was requested on the efficacy of the substance as well as the respective dose and concentration for application. The needs of public relation and an information campaign have been explicitly mentioned by the experts. The meaningfulness of one's actions must be made clear and communicated.

**Table 4:** Evaluation of the respective control measures and identified needs for clarification from the expert interviews made in Germany.

Control measure	Evaluation	Quotation
**Evaluation of the respective control measures**		
EO	Less convincing measure and hardly practicable implementation perspective	It won't work to ask an older woman to bring a bottle of lavender oil and always put a drop inside… But I do have my cent in my wallet Everything that costs money will not be made
Bti	Is largely rejected	I mean, if you can’t do it in a biological way, then you shouldn’t do it at all. We have a huge loss of songbirds anyway
Eurocent coins	High potential for implementation; however only a part of the cemetery visitors seemed to be motivated	People get rid of the stuff they unnecessarily have in their wallets

Topic	Quotation	
**Identified needs for clarification**		
Public relation and information	This requires a clever, broad-based image campaign, and educational work must be carried out. But of course this is a tough undertaking If I transport this accordingly in the media, then the willingness of people will also increase. Especially in the cemetery area I have to inform the older people and they have to be informed with normal media like newspapers and not over the internet. A 70-year-old grandmother who puts a bouquet on her husband's grave then read in the newspaper that she had to put a cent in it, and then she does	
Actual efficacy of the substance	I can’t imagine it working	
Dose and concentration	How many coins in what time intervals?	
Side effect of copper coins e.g. on cut flowers, soil, planting, other organisms such as birds and squirrels	If then the bouquet lasts only one or two days instead of one week…	

Quotations indicate direct remarks from the CATI partners. EO: Essential oil. Bti: *Bacillus thuringiensis israelensis.*

The majority of the experts showed a high willingness to prevent and control vectors (Table 5). They felt responsible for the topic and a major motive to support the measures was the health of the employees. Besides the Eurocent coin method, the avoidance of standing water in e.g. vessels was evaluated very high regarding effectiveness and feasibility. In order to increase the implementation of the measures, an official statement from the local authorities is mandatory from the experts’ perspective. However, there are few voices that raised concerns regarding the control measures. Especially experts from rural areas neglected the topic and trivialized the problem. As a consequence, they were not convinced by the measures and their enforcement. They felt no real threat at the moment but agreed to consider the topic when a real risk is present.

**Table 5:** Willingness to prevent and control vectors (expert interviews).

PRO: High willingness Majority	CONTRA: Low willingness Few voices
Feel responsible for this topic, especially employee health as a major motive for acceptance of control measures	Topic is not taken seriously, but trivialized; especially in rural communities Problem is seen as private matter
Avoidance of standing water and copper coin method is considered feasible	Measures presented are considered unenforceable
The participation of employees and colleagues is rated as high	Only in case of real risk measures could be considered
Official statement from local authorities is expected and considered to be helpful	Great agreement for education at the appropriate time of a real risk event

### Telephone survey

**Figure 3:**
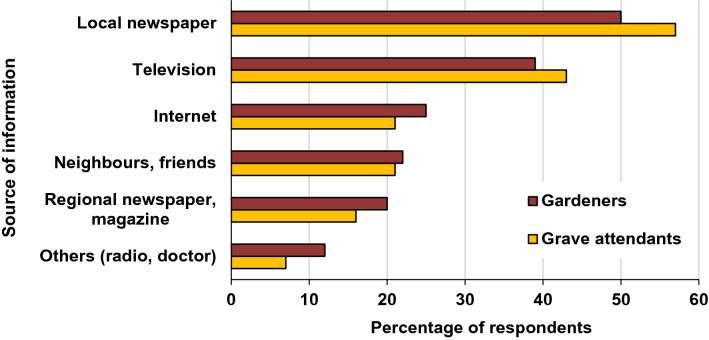
Sources of information for the knowledge that exotic mosquitoes exist in Germany. The data basis is a CATI survey with 257 gardeners and 150 grave attendants in the federal states of Hesse, Baden-Württemberg and Rhineland-Palatinate in Germany.

The majority of the respondents of both groups, gardeners (69%) and grave attendants (65%), have already heard about exotic mosquito species in Germany. For those respondents, the local press and television apparently play major roles as a source of information (Fig. 3).

**Figure 4:**
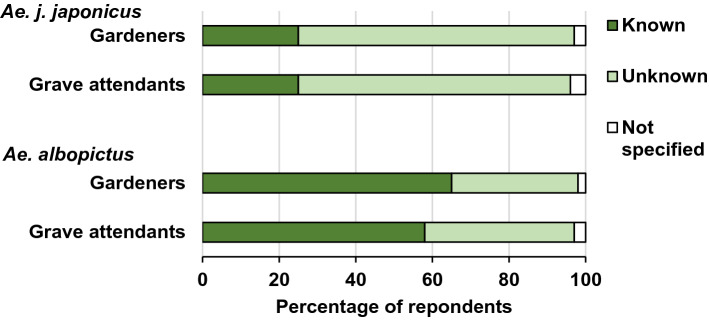
Knowledge of *Ae. j. japonicus* and *Ae. albopictus*. The original question was “These new invasive mosquitoes are mainly two species: The Asian bush mosquito and the Asian tiger mosquito. Have you heard about them?” *Source*: CATI survey with 257 gardeners and 150 grave attendants in the federal states of Hesse, Baden-Württemberg and Rhineland-Palatinate in Germany.

*Aedes albopictus* is much better known than the *Ae. j. japonicus* (Fig. 4). Only a quarter (25%) of respondents in both groups have heard of the latter. In case of *Ae. albopictus*, 65% of the gardeners and 58% of the grave attendants have heard about them (Fig. 4).

**Figure 5:**
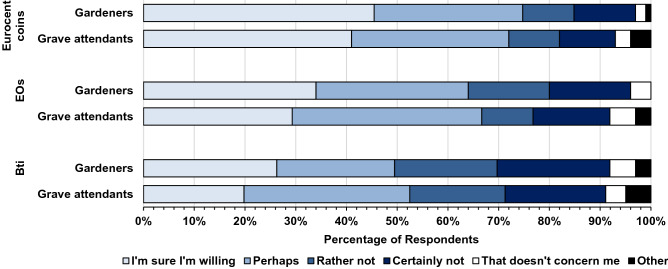
Willingness to implement the respective control measures, such as Eurocent coins, EOs, and Bti tablets. *Source*: CATI survey with 257 gardeners and 150 grave attendants in the federal states of Hesse, Baden-Württemberg and Rhineland-Palatinate in Germany. The category “Other” contains the answer “Don’t know” and the information denied option. EO: essential oil. Bti: *Bacillus thuringiensis israelensis*.

Figure 5 shows that there is a high willingness of the respondents to implement the copper coin method or EOs: 74% of gardeners and 72% of grave attendants are willing to apply Eurocent coins. Clove EO would be implemented by 66% and 64% of the respondents, respectively, while Bti has the lowest acceptance (53% and 49% would use Bti in form of tablets).

## Discussion

We conducted interviews and asked for the grade of acceptance of control measures and willingness for application among potential users (experts, grave attendants and gardeners). In the interviews, we presented conventional Bti, the oviposition-deterrent and larvicidal clove EO and the larvicidal, but not oviposition-deterrent Eurocent copper coins as potential control insecticides against exotic mosquito species. Interviewees clearly preferred Eurocent copper coins as control substance over Bti and clove EO. Also, we asked respondents whether or not they have heard of the exotic mosquitoes *Ae. j. japonicus* and *Ae. albopictus*.

### Knowledge on exotic mosquitoes

During CATI interviews, the majority (> 50%) of gardeners and grave attendants (Fig. 4) specified that they have heard about *Ae. albopictus* being present in Germany while only a quarter of respondents had heard of *Ae. j. japonicus* (Fig. 4). In contrast, *Ae. j. japonicus* is the most wide-spread exotic mosquito in Germany with larger geographical distribution than *Ae. albopictus*^50^. This may be due to the higher medial presence of *Ae. albopictus* and its vectorial competence for more human pathogens than *Ae. j. japonicus*^51^. The knowledge on exotic mosquitoes is mainly gained from local newspapers and television (Fig. 3). These results can facilitate the choice of information channels for awareness-raising and promotion of control measures. This is important information for the design of educational campaigns accompanying mosquito control, because the media used for mosquito awareness-raising does not necessarily lead to a reduction of breeding habitats as shown for print messages and reduction of *Ae. albopictus* breeding habitats in Washington DC, USA^52^. At the same time, the expert interviews showed that profound awareness of the presence of exotic mosquitoes (and other neobiota) exists.

### Support of control options

One human factor in mosquito-borne diseases outbreaks is the will to apply control and protection measures. A survey conducted in North Carolina, USA, has shown that respondents in three counties prefer to wear repellents (75%) followed by removal of potential breeding habitats (67%) to protect themselves against mosquitoes, while 31% have applied or plan to apply insecticides at their residential yards^53^. These results are in line with results of our expert interviews that preventive measures like breeding source reduction are preferred over insecticide application (Table 5). However, in the Perth area, Western Australia, a survey among residents identified a knowledge gap of respondents regarding correct breeding habitat identification and mosquito biology^54^, which complicates the realisation of source reduction measures. This study assessed to which extent mosquito breeding, mainly of *Aedes notoscriptus*, at private residences contributes to the overall mosquito problem (nuisance and disease transmission) and what residents think and do about mosquito control: Sixty percent thought that mosquito control is a governmental task rather than the duty of private persons^54^. In our study, more than 50% of grave attendants and gardeners will or will perhaps apply the suggested substances for mosquito control as private persons. In Germany, questionnaire surveys helped in identification of *Anopheles plumbeus* breeding sites and subsequently improved the efficiency of expert-based monitoring activities, albeit respondents were not efficient in identifying potential breeding habitats of this species on their residential yards^55^. Thus, preventive removal of breeding habitats by private persons may be difficult due to unidentified containers and likely requires extensive training.

For the group of experts, the motivation for supporting and actively applying mosquito control is the protection of the personal health and safety in the workplace. In this study, a temperate mosquito (*Ae. j. japonicus*) was used as a model in a high-income country without area-wide endemic disease outbreak. Thus, disease-transmission and control measures are hypothetical. Control activities in this setting should be routine activities against mosquito nuisance and for disease prevention rather than emergency measures in response to a disease outbreak^22^. In the Athens metropolitan area, Greece, perceptions of citizens and experts were assessed and both groups rated health impacts, related to *Ae. albopictus*’ presence in that area, higher than nuisance impacts (more than 70% of citizens found health impacts “highly important”)^56^. The perception of nuisance and health impacts may change setting-dependently and should be assessed routinely during surveillance and monitoring.

### The need for accepted larvicides usable at private property

In Germany, the only authorised insecticide for use on private ground by non-professionals is Bti in form of tablets which can be directly applied or solved in water for spraying^57^. Unluckily, this is the least preferred control measure during the surveys presented here. In expert interviews and CATIs, we described its formulation as a tablet. Bti is a mixture of endotoxins naturally produced by the bacterium *Bacillus thuringiensis israelensis*. Nevertheless, it is perceived negatively as “chemical” and expected to harm non-target organisms. Whether this is due to the formulation or a general reservation remains unclear from our data collection but another formulation may be a solution. Thus, this shows the importance of developments of publicly accepted larvicides for use by non-professionals on their private property. Bti is considered to be dipteran-specific, but newer publications show its non-target and trophic effects^14,58,59^, albeit other studies suggest no side-effect of Bti^60–62^.

### Eurocent coins and clove EO: effectiveness and toxicity

We have tested eleven substances for their repellent effect for oviposition of *Ae. j. japonicus* in the field. The best repelling substance (clove EO) was then tested in the lab for its acute toxicity against first instar larvae. In addition, we tested the toxicity of Eurocent coins in the lab since it was the preferred method in the surveys. We presented clove EO, Eurocent coins and Bti as effective larvicides during interviews and surveys. Thereby not accounting for the low toxicity of Eurocent coins and the high concentrations of clove EO needed to kill first instar larvae, the most sensitive larval stage in order to find out which factors other the efficiency facilitate the decision of experts and citizens to apply control methods.

We could show that copper ions solved from Eurocent coins are toxic against first instars but 100% mortality was not reached. But 100% mortality may also not been necessary in order to reduce a population as shown in a modelling study for *Ae. j. japonicus*^38^. Copper ions originating from copper electric cables^63^, copper spray^46^, copper sulfate pentahydrate^64^, and vase liners^45^ showed larvicidal activity, which makes this control substance easy available since not a specific copper ion source seems to be needed. Copper ions are also toxic against other mosquito species like *Ae. albopictus *^63^, *Culex pipiens* (*s.l.*)/*Culex torrentium*, and *Aedes aegypti*^46^ which is a prerequisite for IVM targeting multiple mosquitoes. Copper can harm human health and non-target water organisms.

The oviposition-deterrent activity of clove EO could be found in other mosquito species (*Anopheles stephensi*, *Anopheles subpictus*, *Ae. aegypti*, *Ae. albopictus*, *Culex quinquefasciatus*, *Culex tritaeniorhynchus*) as well^65^. Additionally, clove EO targets not only the egg stage as oviposition-deterrent and the larval stages as a larvicide against *Ae. j. japonicus* (this study), *Ae. aegypti* and *Cx. quinquefasciatus*^66^. Clove EO was also shown to be a potent adult repellent in laboratory and field settings against *Ae. aegypti*, *Aedes cinereus* and *Aedes communis*^67^. This is a pre-feature for IVM targeting multiple stages and mosquito species. Clove EO’s LC_50_ values calculated here were comparable to mortality effects in an insecticide susceptible *Anopheles gambiae* (*s.s.*) laboratory strain (LC_50_ = 17 µg ml^−1^)^68^. Clove EO’s LC_50_ values for *An. stephensi*, *An. subpictus*, *Ae. aegypti*, *Ae. albopictus*, *Cx. quinquefasciatus* and *Cx. tritaeniorhynchus* after 24 h range from 51 to 72 µg ml^−1^^65^ and for *Ae. aegypti* and *Cx. quinquefasciatus* from 92 to 124 mg l^−1^^66^. Such differences may account to extraction methods (bud, leaf or seed of clove), mosquito species and stage, solvent use as well as seasonal and geographical variation in clove metabolites. Other tested substances showed contradictory effects on egg-laying between the two seasons. This may be due to a changed oviposition behaviour in the course of a year or the different lengths of the experimental time periods. During the latter period, which was longer, eggs may have been missed since larvae already hatched.

### Eurocent coins and clove EO: considerations of other factors influencing their application

The public perception of Bti, Eurocent coins and clove EO adds important information to the efficiency study. Apparently, factors besides efficiency play an important role in the willingness of experts and citizens to carry out insecticide application: Factors pinpointed by survey participants were accessibility, cost, and formulation of alternative insecticides. Eurocent coins are easier to apply because there is no need for frequent replacement, while clove EO treatment needs to be renewed. Eurocent coins take effect after being incubated in water since ions need to be solved. Since *Aedes* mosquitoes lay eggs only in water-filled containers, the Eurocent coins are in water at least once and ions are present even after a dry period. It has to be pointed out to potential users that this method requires not cleaning containers to avoid removal of ions. The easy access of the low-value Eurocent coins by private persons is clearly perceived as an advantage (Table 4).

To transfer these results to field conditions, however, further research is needed. Application frequency and amount as well as the effect on older larval stages need also to be assessed. Provision of knowledge of these prerequisites is also demanded by experts (Table 4).

Based on this study, the material costs of Eurocent coins as a potential control substance can be very low in the case that they can be reused after larvicidal application, e.g., in publicly inaccessible gardens (Supplement S5) but it also can cost many times more than Bti. Therefore, Eurocent coins as potential control substance may only be possible in the context of private, non-commercial, and locally restricted use. A large-scale use is likely hampered by the provision of a sufficient amount of Eurocent coins. Altogether, promotion of Eurocent coins for larvicidal use should carefully considerate and point to this specific scope of application.

### Considerations for the planning and implementation of IVM measures

The implementation of effective and consistent control measures against invasive mosquito vectors is difficult on privately owned land^24,28,69,70^ due to limited access by officially tasked authorities. An alternative is the application of the control method directly by the owner, user or inhabitant. However, to assure acceptance and support of the respective measures as well as a reliable application, thorough information campaigns are necessary^25^.

IVM includes the application of different tools, e.g., insecticides, for the reduction of vector populations. Our study clearly shows that the decision on what tools will be used needs not solely be justified on the insecticide’s toxic effectiveness but also on the willingness of potential users to apply them.

The arrivals of invasive mosquito species and their related pathogens not only pose trouble to the health sector but also to legislation. The legal ranking of the mosquito as invasive species and/or disease vector must be taken into account. In addition, risk assessments for spatial hotspots and vector capacities are needed to justify measures.

*Aedes j. japonicus* is a minor vector of less pathogens compared to *Ae. albopictus*^51^. However, since we did not strictly differentiate between exotic mosquitoes in the interview and the CATI, some results like the general will to apply which insecticide of this study are transferable to other exotic container-breeding mosquitoes. Different non-native species of the genus *Aedes* are present in Germany. In the federal state Hesse, three species occur, *Ae. j. japonicus*^71^, *Ae. koreicus*^72^, and *Ae. albopictus*^73^. For this reason, IVM targeting multiple mosquito species should be a priority. Thus, future work should be dedicated to in situ toxicity testing of Eurocent coins against the exotic mosquitoes mentioned above. In addition, non-target effects should be tested since trade-offs between environmental health and public health should be minimised, especially in times of insect biomass loss^74^.

Certainly, assessment to show changes on population level regarding resistance formation as well as future work on chronic potency, temperature stability, application frequency, spill-over effects on soil organisms, and field evaluation is required to finally approve Eurocent coins and clove EO as alternative insecticides. This goes also in line with the requirements for insecticide approval mentioned during expert interviews.

This study shows that reservations made by potential insecticide users need to be considered when testing and developing insecticides. Social surveys conducted at an early time of insecticide development may lead to shorter product development time because only preferred insecticides can be kept track with, which is time and resource-saving. In addition to the substances in this study, synergistic use of control tools can be evaluated, e.g., mass trapping of adults can be included^38^ supplemented by educational campaigns like the Citizen Action through Science approach^75^. To sum up, this study contributes to our knowledge of social factors influencing the application of insecticides by experts and two potential user groups (gardeners and grave attendants). This information can be implemented as a social factor in modelling the efficiency of mosquito control methods. Furthermore, this study presents social factors, potential new insecticides, and toxicity data valuable for control-measure planning.

## Supplementary information

Supplementary Information.
