# Supplementary material for: Knowledge on exotic mosquitoes in Germany, and public acceptance and effectiveness of Bti and two self-prepared insecticides against Aedes japonicus japonicus
Source: Sci Rep. 2020 Nov 3;10:18901. doi: 10.1038/s41598-020-75780-5 (PMC7641113; doi:10.1038/s41598-020-75780-5)
Supplement: Supplementary file 1 — Supplementary Information. [file 41598_2020_75780_MOESM1_ESM.docx]

Supplementary Material

Knowledge on exotic mosquitoes in Germany, and public acceptance and effectiveness of Bti and two self-prepared insecticides against *Aedes japonicus japonicus*

Friederike Reuss ^1,^*, Aljoscha Kreß ^2^, Markus Braun ^2^, Axel Magdeburg ^1,2^, Markus Pfenninger ^1,3,4^, Ruth Müller ^2,5,♥^, and Marion Mehring ^1,6,♥^

^1^ Senckenberg Biodiversity and Climate Research Centre (SBiK-F), Georg-Voigt-Straße 14-16, D-60325 Frankfurt am Main, Germany

^2^ Institute of Occupational Medicine, Social Medicine and Environmental Medicine, Goethe University Frankfurt, Theodor-Stern-Kai 7, D-60590 Frankfurt, Germany

^3^ LOEWE TBG (Translational Biodiversity Genomics), Senckenberganlage 25, D-60325 Frankfurt am Main, Germany

^4^ Institute of Organismic and Molecular Evolution (iOME), Johannes Gutenberg University, Gresemundweg 2, D-55128, Mainz, Germany

^5^ Institute of Tropical Medicine, Nationalestraat 155, B-2000 Antwerp, Belgium

^6^ ISOE – Institute for Social-Ecological Research, Biodiversity and People, Hamburger Allee 45, D-60486 Frankfurt am Main, Germany

***** Corresponding author: friederike.reuss@senckenberg.de; Tel.: +49 (0)69 7542 1813

^♥^ These authors contributed equally to this work

Contents:

Supplement S1: Semi-structured interview guide for expert interviews.

Supplement S2: Questionnaire for CATI survey.

Supplement S3: Egg counts of the field oviposition deterrence experiment.

Supplement S4: Regression equations and R^2^ values of copper experiment 1.

Supplement S5: Photographical assessment (on day 30) of Eurocent coins after experimental use.

Supplement S6: Photographical assessment of cut roses without Eurocent coin treatment.

Supplement S7: Photographical assessment of the effect of Eurocent coins on cut roses.

Supplement S8: Regression equations and R^2^ values of toxicity tests.

**S1**: Semi-structured interview guide for expert interviews

• Short project presentation and introduction

*... we are currently conducting interviews with employees of cemetery administrations, green space offices and cemetery nurseries. It's about new mosquito species, which are spreading more and more in Germany. and can be found especially on cemeteries and in allotments. The aim is to find out which measures for prevention and combat are possible with the help of local authorities and population.*

- - Could you please elucidate your professional function and to what extent you are dealing with cemeteries and/or allotments.

• Awareness of the Asian bush mosquito

- - Have you ever heard of new mosquito species spreading in Germany before?
    - If so, do you know what species of mosquitoes they are?
- *If necessary, clarify that there are different mosquito species: Asian bush mosquito and Asian tiger mosquito and that the study concentrates on the Asian bush mosquito.*
  - - What does the interviewee know about this?
    - When did he/she first hear about it? Where and how was it reported?
    - What is (probably) the problem with these mosquitoes? *Let it be briefly explained*
- ***Focus on Asian bush mosquito;*** *short explanation (occurrences: Cemeteries, allotments, development of larvae in standing water in small vessels, watering cans and barrels, transmission of diseases like Japanese Encephalitis and West Nile Virus)*
  - What does these information trigger at the interviewee? What does he/she have in mind?
  - Has the interviewee already encountered such mosquitoes or mosquito larvae somewhere?
  - If so: where, when, to what extent, how often, in which months? What did interviewee notice?
- *Let explain, ask!*

• Control measures for the Asian bush mosquito

*Explain: In the meantime, various scientists are working on the problem of the Asian bush mosquito in Germany. From the scientists' point of view, it is very important to combat the Asian bush mosquito effectively in order to prevent its spread.*

*This project is primarily concerned with environmentally friendly and sustainable precautionary and control measures. The addition of essential oils (lavender, clove) or a copper cent piece to the vessels with standing water, e.g. flower vases, holy water bowls, plant pots etc. is currently being tested in field trials using cemeteries or allotment gardens as examples. Laboratory tests have already shown good results.*

- - First reactions: what does the interviewee think of such measures?
  - How does the interviewee assess whether such measures can be implemented in cemeteries / small gardens?
    - What are the advantages? What opportunities does the interviewee see?
    - What are the reasons against it? What difficulties or barriers does the interviewee see?
    - How does the interviewee see the willingness of the cemetery staff / gardeners to participate?
    - ... and how willing are the keepers of a grave / allotment gardeners to participate?
  - How does the interviewee evaluate the specific possible measures:
    - Essential oils / cent pieces / avoidance of standing water
    - What differences do you see in the application possibilities? Which measures are more promising than others? Why?

*Another possibility would be prevention: standing water in vessels or rain barrels would have to be avoided (at least temporarily).*

- - What does the interviewee think of this measure?
  - How does the interviewee assess whether such a measure can be implemented in cemeteries / allotments?
    - What speaks for it? What opportunities does the interviewee see?
    - What speaks against it? What difficulties or barriers does the interviewee see?
    - How does the interviewee see the willingness of the cemetery staff / gardeners to participate?

- ... and how the willingness of the keepers of a grave / the allotment gardeners to contribute to this?

*Another possibility would be the extensive use of insecticides, e.g. BTI*

*(BTI is dropped in floodplains by helicopter as ice balls. In a cemetery one would rather mix BTI in water and pour it into water containers.)*

- - What does the interviewee think of this measure?
  - How does the interviewee assess whether such a measure can be implemented in cemeteries / allotments?
    - What speaks for it? What opportunities does the interviewee see?
    - What speaks against it? What difficulties or barriers does the interviewee see?
    - How does the interviewee see the willingness of the cemetery staff / gardeners to participate?

• Potential help from the offices / cemetery nurseries

- - How does the interviewee see the role of municipal offices / cemetery gardening?
  - To employees of the green area offices / cemetery administrations: Who would be responsible for this from the interviewee’s point of view within the offices?
    - For cemeteries
    - For allotments
  - What would be the requirements for the relevant staff to support measures to combat Asian bush mosquitoes?
    - What are the prerequisites for this?
    - Does the interviewee see difficulties? Which, why?
  - What concrete possibilities does the interviewee see for helping to combat this problem?
  - What might this look like? e.g.
    - Information and motivation of customers / display of information flyers
    - Provision of essential oils in the cemetery / small garden?
    - Use of essential oils / cent pieces by the cemetery gardeners / allotment gardeners
    - To cemetery gardener: Sale of essential oils in the shop ...
  - What prerequisites would have to be met?
  - Does the interviewee see difficulties? Which, why?
  - How does the interviewee see the willingness of the carers of a grave to participate?

• Comparison of methods

- - Which of the 4 methods - essential oils / cent pieces / avoid standing water / spraying method (BTI) - does the interviewee consider rather promising, which rather not practicable or to be rejected?
    - Reason for each

• *To those responsible:* Estimates of the number of water vessels in cemeteries

How common do you think are in the cemeteries you care for, vessels of standing water?

- - Estimated, how many of 100 graves have such containers in them, e.g. flower vases, holy water bowls, plant pots?
    - Which types of vessels are particularly common?
    - How does this look during the mosquito season, i.e. between March and October?
  - According to the interviewee, how often is the water exchanged by visitors?
- *To all:*

• Accessibility

- - What possibilities does the interviewee see for making essential oils accessible to users?
    - - Sale in shops
      - Provision at the cemetery / Accessibility?

• Information and education on combating the Asian bush mosquito in cemeteries and allotment gardens

- - How could or should cemetery visitors and allotment gardeners be informed and educated about the topic?
- *First ask openly, then add:*
  - - Press work
    - Municipal newsletters
    - Posters, where?
    - Flyer to display ...
  - What other possibilities does the interviewee see? What ideas exist?
  - What could the responsible employees of the municipality / the cemetery gardeners contribute? What would be realistic?

• End and thanks

**S2**: Questionnaire CATI

**Screening: garden ownership, containers in gardens**

Do you have a garden?

- No  Discontinuation
- Yes, I do.

*If so:* what kind of garden is it?

- A garden right next to the house
- A garden in an allotment garden
- A garden on a private property
- Other: What kind?

What do you have in your garden?

*Yes/No*

- A water barrel with cover
- A water barrel without cover
- A pond
- A well
- A bird bath
- Empty, old flowerpots or cachepots in which water can stand
- Flower pots/plant pots with saucer
- Flower pots/plant pots without saucers

*Min. one of the first 7 answers with yes, otherwise discontinuation*

**Screening: grave care, vessels on grave**

Do you regularly maintain a grave or several graves in the cemetery yourself?

- No  Discontinuation
- Yes, I do.

How many graves do you maintain?

- Number:

Which of the following items are regularly on the grave/ on at least one of the graves?

- A flower vase / flower plug-in vase  if yes, see below
- A plant bowl with coaster
- A plant bowl without coaster
- None of it

*Min. one of the first 2 answers with yes, otherwise discontinuation*

*if yes: does the flower vase always stands on the grave or only as long as it is filled with flowers?*

- Always on the grave
- Just as long as it's filled with flowers

**After the screening: Only to grave attendants**

How often do you visit the grave or graves in summer on average?

- Once or several times a week
- Two to three times a month
- Every one to three months
- Rarer

Is there standing water in a container on or behind your grave at least from time to time?

- Yes, in a flower vase
- Yes, in the coasters of plant bowls
- Yes, in other jars, which one?
- No

Do you have objects stored behind the grave or graves that you use for the grave, e.g. a vase or watering can?

*Yes/No*

Do you sometimes have to struggle with musty, stinking water on your grave?

*Yes/No*

When you think about this summer, have you noticed any mosquitoes in the cemetery?

*Yes/No*

*If so,* did you feel harassed by mosquitoes in the cemetery this summer?

*Scale: Yes, very / to some extend / a little / no, not at all*

All of you who were *very/ to some extent annoyed*: What kind of mosquitoes did you notice? (Multiple answers)

- Normal mosquitoes
- New mosquitoes which have not been around for long
- Not sure

Have you ever seen mosquito larvae or breeding mosquitoes in standing water at the cemetery?

*Yes/no in each case*

- In the public standpipes / wells
- In plug-in vases
- In coasters
- In a watering can
- In other vessels, which one?

**After the screening: To garden owners**

How do you use your garden most of all? (Multiple answers)

- As a kitchen garden: e.g. fruit, vegetables, herbs
- As an ornamental garden: flowers and perennials
- For recovery
- For children to play
- For other things, which?

How big is your garden?

- Square meters:

Mosquitoes are often an annoying topic in gardens. What about you when you think of this summer: how much did mosquitoes bother you in your garden?

*very harassed / to some extend / a little / not harrassed*

All of you with very/what annoyed: What kind of mosquitoes did you notice? (Multiple answers)

- Normal mosquitoes
- New mosquitoes which have not been around for long
- Not sure

Does standing water occur in your garden sometimes, e.g. in containers or coasters?

*Yes/No*

*If yes:* does the standing water sometimes get musty and start to stink?

*Yes/No*

Everyone: Have you ever observed mosquito spawn or mosquito larvae in standing water in your garden?

*Yes/No*

- In the water barrel / rain barrel
- In coasters of tub plants
- In flowerpots
- In the watering can
- In the pond / fountain
- In other vessels, which one?

**To all: Topic mosquitoes**

In Germany, there have been new mosquito species in recent years, which are increasingly spreading. Have you ever heard of new mosquito species in Germany?

*Yes/No*

*If so*, where did you hear about it?

- In the local newspaper
- In the regional newspaper or magazine
- On television
- On the internet
- Through conversations with neighbors, friends, acquaintances
- By other means, how?

These new invasive mosquitoes are mainly two species: the Asian bush mosquito  and the Asian tiger mosquito. Have you heard of them?

*Yes/No*

- Asian bush mosquito
- Asian tiger mosquito

If at least one of them is yes: Have you already encountered such mosquitoes or mosquito larvae somewhere?

*Yes/No*

- Asian bush ornament, if yes: where?
- Asian tiger mosquito, if yes, where?

The following is about the **Asian bush mosquito**. It occurs a lot in allotments and cemeteries, because its larvae develop in standing water, e.g. in small vessels, coasters and water barrels. This mosquito species has been on the rise, so far especially in southern and western Germany. It can transmit diseases such as Japanese encephalitis or the West Nile virus.

When you hear this information about the Asian bush mosquito, what reactions does it trigger in you? To what extent do you agree with the following statements? (Rotate)

*Quad scale: agree completely / agree rather / disagree rather / disagree completely".*

- I think that is a serious threat.
- I think the dangers of such mosquitoes here in Germany are exaggerated.
- I have a number of unanswered questions that need to be answered.
- I think there is an urgent need to raise awareness of this mosquito species.
- I would be very interested to know what can be done against this mosquito species.

To what extent do you feel affected yourself by the health hazards that can emanate from the Asian bush mosquito?

*Quad scale: very affected, to some extend affected, a little affected, not affected*

*Allotment gardeners only:*

In order to prevent the spread of Asian bush mosquito, small-scale gardeners are particularly in demand. There are various ways of combat. One can e.g. avoid containers with standing water, cover the water barrels, fill up the coasters with sandy gravel or fight these mosquitoes with different products, which are given into the water.

I will now read out various control measures to you and ask you to tell me to what extent you would be willing to take the specific measures. (Rotate)

*I am certainly willing / possibly / rather not / definitely not / that does not concern me*

- Empty coasters / small vessels regularly
- Fill coasters with sandy gravel
- Do not use containers with standing water at all
- Always cover the water barrels
- In the pond: deploy fish/amphibians which eat the larvae

Various measures are currently being tested to combat the Asian bush mosquito, some of these are intended to be particularly environmentally friendly.

Here are three examples. To what extent would you be willing to use the following control measures? (Rotate!)

*I am certainly willing / possibly / rather not / definitely not / that does not concern me*

- Place copper coins, i.e. 1, 2 or 5 cent pieces from the wallet into the container. Two to three coins are needed for every 1 liter of jar. These work for 3 to 4 weeks. Then they can be used as coins again.
- Essential oil, e.g. clove oil in the water of the small vessels, coasters, or water-tons drip. Approx. 5 drops to 1 liter of water, the effect lasts about a week.
- Pour tablets with BTI into the water. BTI is a bacterium as larvicide, which fights the larvae biologically. One tablet is enough for one vessel with 50 liters of water and lasts for about 4 weeks. The water can be used for watering.

Please **select two** of the proposed measures that are most likely to be of interest to you:

*All from above, with "I am sure ready / possibly ready"*

- Empty coasters/small containers regularly
- Fill coaster with sandy gravel
- Do not use vessels in which standing water can form at all
- Always cover the water barrels
- In the pond: fish/amphibians feeding on the larvae
- Place the copper cent pieces in the container
- Clove oil dripping into the water
- Pour tablets with BTI into the water

*Only to grave attendants:*

In order to prevent the spread of the Asian bush mosquito, the keepers of graves are in particular demand. There are various control measures. One can avoid e.g. containers with standing water, or empty regularly, coasters with sandy gravel fill up or fight these mosquitoes with different means, which are given into the water.

To what extent would they be prepared to take the following control measures? (Rotate!)

*I am certainly willing / possibly / rather not / definitely not / that does not concern me*

- Do not use vessels, e.g. vases with standing water
- Empty vases and coasters regularly
- Fill coaster with sandy gravel
- Do without coasters completely, in which standing water is formed

Various measures are currently being tested to combat the Asian bush mosquito, some of which are intended to be particularly environmentally friendly.

o what extent would you be willing to use the following control measures? (Rotate!)

*I am certainly ready to do so / possibly / rather not / definitely not / that does not concern me*

- Place copper coins, i.e. 1, 2 or 5 cent pieces from the wallet into the container. Two to three coins are needed for every 1 liter of jar. These work for 3 to 4 weeks. Then they can be used as coins again.
- Essential oils, e.g. clove oil, drip into the water of the small vessels, coasters, or water-tons. Approx. 5 drops to 1 liter of water, the effect lasts about a week.
- Pour tablets with BTI into the water. BTI is a bacterium that fights the larvae biologically. One tablet lasts about 4 weeks. The water can be used for watering.

Please **select two** of the proposed measures which are most likely to be of interest to you:

*All from above, with "I am sure ready / possibly ready".*

- Do not use vessels, e.g. vases with standing water
- Empty vases and coasters regularly
- Fill coaster with sandy gravel
- Do without coasters completely, in which standing water is formed
- Place the copper coins in the container.
- Clove oil dripping into the water
- Pour tablets with BTI into the water

*To all:* Which information channels and media are particularly suitable for you personally for education and information about the Asian bush mosquitoes?

*Scale of 4: very suitable / suitable / less suitable / not suitable*

- Television
- Radio broadcasts
- Local newspaper
- Online edition of the daily newspaper
- Internet
- Free advertising leaflets / weeklies
- Official newsletter / town gazette
- *only to grave attendants:* Church parish letter on the topic of grave care
- Social media, e.g. Facebook ...
- Posting in showcases / notice boards on cemeteries / in allotment gardens
- Postcards to take with you
- Direct mail
- Conversations with neighbors, friends and relatives

May I ask how old you are?

- Years

Sex:

- male/female/divers

What is your highest level of education?

- Still pupil
- Left school without a qualification
- Lower secondary school
- Secondary modern school / commercial school / vocational school without Abitur
- Abitur / vocational diploma / University entrance qualification
- Completed studies (university degree / academic degree)

How many people live in your household?

- Persons

**Thanks and End**

**S3**: Egg counts of the field oviposition deterrence experiment.

**S4**: Regression equations and R^2^ values of copper experiment 1.

|  | Tap water | Rainwater | Deionised water |
| --- | --- | --- | --- |
| pH | y = 0.02640 * x + 7.626  R^2^ = 0.1748 | y = 0.2183 * exp(-0.2481 * x) + 7.417  R^2^ = 0.02336 | y = 1.209 * exp(-0.7042 * x) + 6.666  R^2^ = 0.4006 |
| Conductivity | y = 6.176 * x + 540.7  R^2^ = 0.8569 | y = 1.542 * x + 127.6  R^2^ = 0.8874 | y = 0.3134 * x + 0.8203  R^2^ = 0.8824 |
| Cu^+^ | y = 0.04515 * exp(0.05678 * x)  R^2^ = 0.5982 | y = 0.0813 * exp(0.07152 * x)  R^2^ = 0.7286 | y = 0.1067 * exp(0.09455 * x)  R^2^ = 0.9695 |
| Cu^2+^ | y = 0.04354 * exp(0.05915 * x)  R^2^ = 0.6393 | y = 0.07994 * exp(0.07209 * x)  R^2^ = 0.7282 | Y = 0.1043 * exp(0.09618 * x)  R^2^ = 0.9693 |


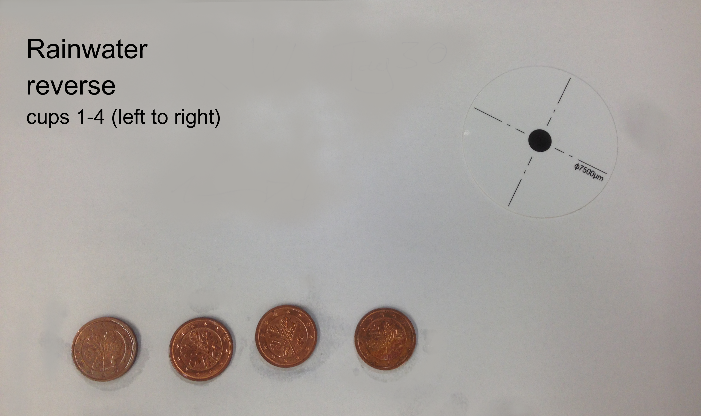

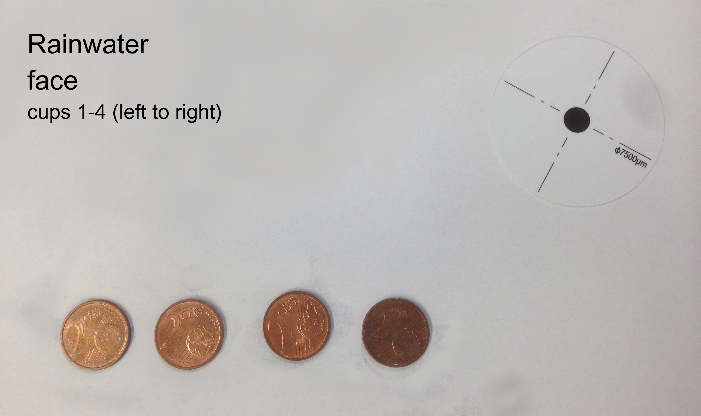

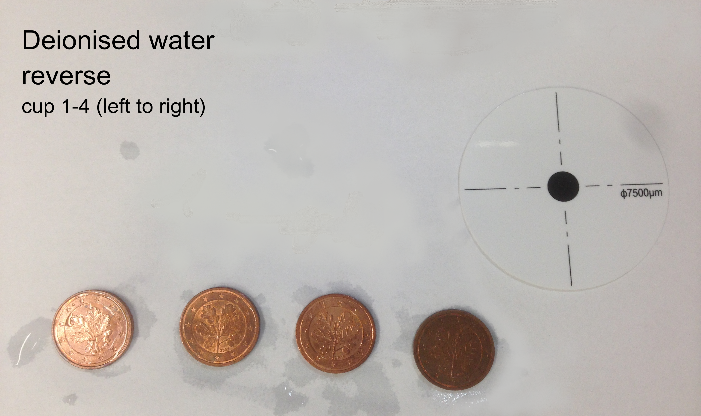

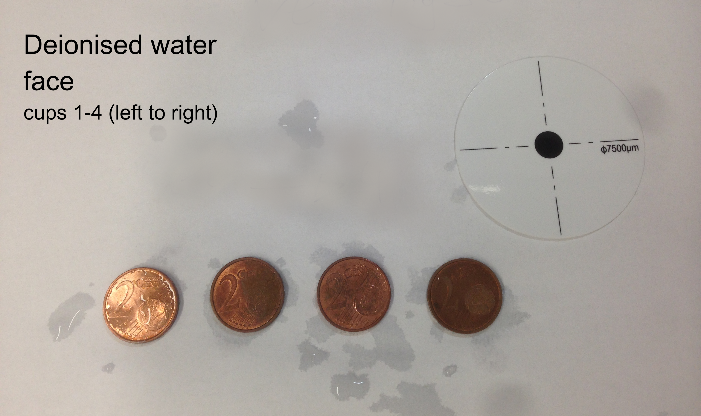

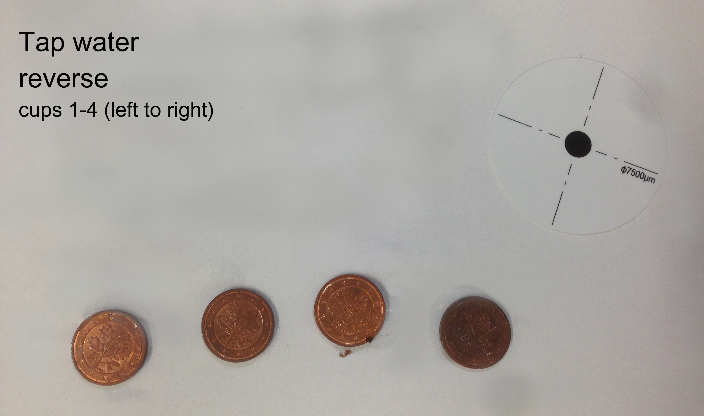
**
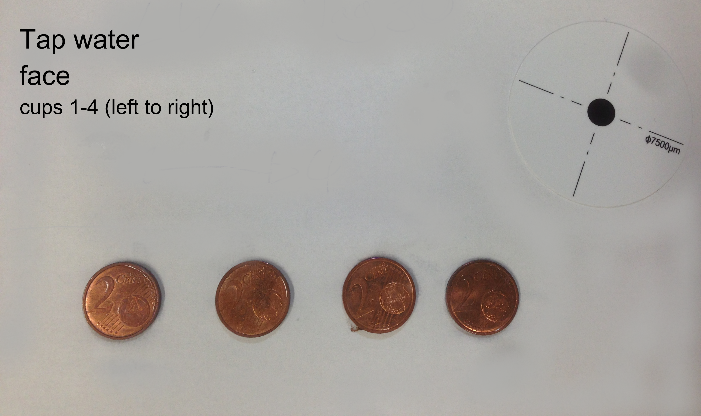
S5**: Photographical assessment of copper coins after experimental use (on day 30)

**S6**: Photographical assessment of cut roses (no copper treatment)


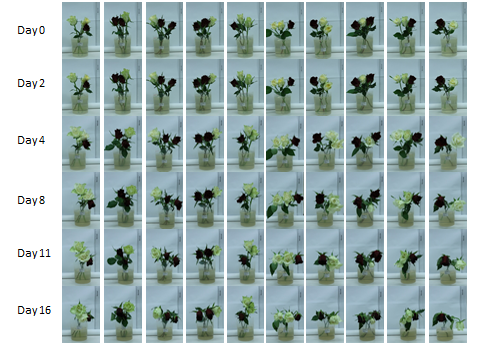


**S7**: Photographical assessment of the effect of cut roses (copper treatment)


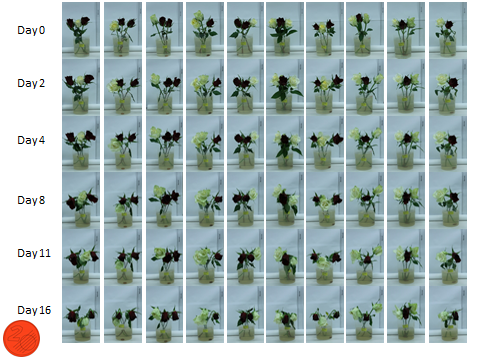


**S8**: Regression equations and R^2^ values of toxicity tests. EO: essential oil.

| Experiment | Equation | R^2^ |
| --- | --- | --- |
| Clove EO Hadamar | y = 100 / (1 + 10 * exp((1.242 – x) * 2.436) | 0.9629 |
| Clove EO Dorndorf | y = 100 / (1 + 10 * exp((1.22 – x) * 1.88) | 0.8559 |
| Copper | y = 100 / (1 + 10 * exp((2.696 – x) * 0.2) | 0.8000 |
